# The relationship between serum total alkaline phosphatase and risk of osteoporosis: a cross-sectional study

**DOI:** 10.3389/fendo.2025.1657631

**Published:** 2025-12-05

**Authors:** Yuang Chen, Yong Zhang, Mao Nie

**Affiliations:** 1 Department of Orthopedic, The Second Affiliated Hospital of Chongqing Medical University, Chongqing, China; 2 College of Public health, Chongqing Medical University, Chongqing, China

**Keywords:** osteoporosis, bone turnover, alkaline phosphatase, cross-sectional study, liver enzymes

## Abstract

**Objectives:**

to extensively investigate the relationship between serum total alkaline phosphatase (ALP) and osteoporosis in a large-scale population.

**Methods:**

people who took routine health examinations between 2019 and 2024 at a teaching hospital were included. The health examination records were reviewed, and data were extracted. The variables included anthropomorphic measurements, laboratory tests, and biochemical indices. Osteoporosis was defined by bone mineral density at the spine and hip. The relationship between ALP and osteoporosis was analyzed by using logistic regression, Receiver Operating Characteristic (ROC) analysis, and restricted cubic spline analysis.

**Results:**

total ALP in serum was significantly higher in osteoporosis than in non-osteoporosis groups(P<0.001). The relationship between ALP and osteoporosis was persistent in multivariate analysis after adjusting for available confounders. There was a non-linear relationship between ALP and osteoporosis; when ALP was greater than 100 IU/L, the relationship became less pronounced. The relationships between ALP and osteoporosis were stronger in younger, female, and metabolically healthy populations with normal liver enzymes. The best cut-off value for ALP to predict osteoporosis was 72IU/L in ROC analysis.

**Conclusions:**

the serum total ALP in serum persistently and negatively relates to the risk of osteoporosis in a general adults population when ALPs are clinic normal. The 72IU/Lof total serum ALP may be considered as a tentative threshold for initiating further bone health counseling in health management for adults. Cohort studies on the application of ALP in the prediction of osteoporosis or osteoporotic fracture are warranted.

## Introduction

Osteoporosis is featured by low bone mass and micro-architectural deterioration of bone tissue with a consequent increase in bone fragility and susceptibility to fracture, which are associated with morbidity, mortality, and low quality of life (1). Because of the increasing prevalence of osteoporosis and osteoporotic fractures, it is considered a significant public health burden worldwide. In a study in China, the prevalence of osteoporosis for men is about 5% and for women is about 20.6%. At the exact time, the prevalence of vertebral fracture was 10.5% (95% CI, 9.0%-12.0%) among men and 9.7% (95% CI, 8.2%-11.1%) among women (2). There is a trend of increasing incidence of all major fracture types (hip, vertebral, distal radial, proximal humerus) with age, with a near exponential increase in hip fracture incidence in men and women beyond 75 years due to osteoporosis (3).

Therefore, it is crucial to find new biomarkers to improve the prevention and management of osteoporosis. Osteoblasts produce bone alkaline phosphatase (ALP) during bone formation. An important role of ALP is to inactivate pyrophosphate, an inhibitor of mineralization (4). Slightly less than 50% of circulating ALP is bone-derived. The remainder which mainly originates from hepatocytes is called liver ALP. In the absence of cholestatic liver disease, the value of total ALP above the normal range can arguably be considered as the increase of bone ALP, Besides, Bone ALP was found highly correlated to total ALP in normal people, osteoporosis patients and post-menopause people (5). Therefore, both serum total ALP and bone ALP could bring valuable information for the early detection of loss of bone mass or the osteoporosis (6), Because total ALP is more accessible than bone ALP, especially in health examinations and population level, it was often used as an alternative to bone mass testing in epidemiology studies. In NHANES studies, one showed that elevated serum total ALP was significantly associated with low bone mineral density in the femoral neck and lumbar spine. That elevated serum total ALP was associated with an increased prevalence of osteoporosis (7). The other study showed that serum total ALP was negatively associated with lumbar bone mass density among young adults (8). A retrospective study conducted at King Fahd Hospital, Khobar University, suggested that serum total ALP levels were lower in individuals with osteoporosis than in normal individuals and were negatively correlated with bone mass density (9). ALP and low-density lipoprotein cholesterol, as well as a history of hyperlipidemia, were also found to be negatively correlated with bone mass density (10). In postmenopausal women, ALP was significantly lower among healthy controls, negatively related to bone mass density, and osteoporosis (11). In patients with type 2 Diabetes, serum levels of ALP were negatively correlated with bone mineral density at three sites in men and with total lumbar bone mineral density in women (12).

Current research on the relationship between serum total ALP and osteoporosis risk remains insufficient and controversial. The paired relationships between total ALP, bone ALP, and bone mass at different sites have shown discordance in various studies (13), primarily due to self-reported data, sample size, lack of diversity, and failure to incorporate clinical lifestyle data. Furthermore, total ALP can be confounded by several factors, including gender, age, weight, vitamin D, and calcium. It may not be specific for bone turnover in some cases of concomitant diseases, such as liver disease, Paget’s disease, renal disease, and thyroid disease (14), which complicate the situation when using total ALP as a marker in osteoporosis studies and prevention.

To further clarify the role of serum total ALP as a marker for bone health in the context of a complex network of bone metabolism, we utilized health examination data to explore the relationship between total ALP and osteoporosis.

## Methods

### Study design

This was a cross-sectional study. Routine health check-up records from the second hospital affiliated with Chongqing Medical University were used. This study was approved by the Ethics Committee of the Second Hospital, affiliated with Chongqing Medical University (No. 262-2022).

### Participants

The participants were healthy individuals who underwent their routine health examinations between 2019 and 2024. People who 1) were over 20 years old, 2) had blood ALP tests, and 3) had bone dual x-ray examinations were eligible for inclusion. Those with missing data were excluded.

### Data retrieving

Databases were queried to obtain eligible subjects. All physical examinations and serum biological tests were conducted and recorded by standard procedures by qualified personnel at this teaching hospital. For duplicated records with the exact identification, the one with the most recent date was retained.

### Definition of abnormal indices or diseases

Lumbar spine and hip were measured by DPX Bravo DXA scanners (GE Healthcare, WI, USA). According to the WHO recommended criteria (15), osteoporosis is diagnosed for postmenopausal women and men age 50 and older when the bone mass decrease was more than 2.5 reference standard deviation (SD); and for females prior to menopause and in males younger than age 50, bone mass decrease was more than 2 SD is defined as”osteoporosis”in this study. The bone mass scanner diagnoses osteoporosis automatically. Fatty liver disease was diagnosed by abdominal ultrasonography, as fat accumulation exceeded 5% of hepatocytes.

All the laboratory values were determined according to the standard laboratory procedures. According to the suggestions of the Working Group on Obesity in China (WGOC), Body Mass Index(BMI) cutoff values for overweight and obesity were 24~28- and >28, respectively. Furthermore, according to the consensus and criteria set up by the American Association for the Study of Liver Diseases (AASLD) and the European Association for the Study of the Liver (EASL)or ATP III guideline or the clinic diagnosis standards in local laboratory: high Systolic Blood Pressure(SBP) was defined as ≥130 mmHg; high Diastolic Blood Pressure(DBP) >85 mmHg; high Glucose(GLU) ≥ 100 mg/dL (≥5.6 mmol/L), high Glycated Hemoglobin(GHB) ≥ 5.7%; high Total Cholesterol(TC)> 240mg/dl (>6.2 mmol/L); high Triglyceride(TG) ≥150 mg/dL (≥1.70 mmol/L); low High-Density Lipoprotein(HDL) <40 mg/dL(<1.0 mmol/L); high LDL(Low-Density Lipoprotein) > 130mg/dl (> 3.4 mmol/L); high Uric Acid(UA) >7mg/dl (>420 µmoL/L); high Aspartate Aminotransferase(AST)>40UI/L; and high Alanine Aminotransferase(ALT) >40UI/L. The reference interval of ALP is 45-125IU/L for male and 35-100IU/L for female with age < 50 and 50-135IU/L for female age >=50 in our hospital.

### Statistical analysis

In descriptive analysis, continuous variables were expressed as means ± standard deviations, and categorical variables were expressed as numbers and percentages. Comparisons among different groups were conducted using the t-test, analysis of variance (ANOVA), or the Chi-squared test. Five Logistic regression models were used to determine the odds ratio (OR) and 95% confidence interval (CI) to examine the association between ALP and osteoporosis. Models were arbitrarily built by introducing a group of covariables sharing some common features. Model 1 was the crude model, no covariates were introduced; model 2, we adjusted the age and sex, which are the basic most important characteristics of a person; model 3, we adjusted BMI and waist-hip ratio, which represent the body shape; model 4, blood tests were introduced to see what effects of metabolic parameters can have; model 5, we introduced liver related parameters related to see if liver functions can affect the results. We introduced these variables because they were statistically different between normal and osteoporosis.

Furthermore, restricted cubic spline analysis was performed to examine the nonlinear associations between ALP and osteoporosis by introducing four knots positioned at the 5th, 35th, 65th, and 95th percentiles of ALP. Besides, the Receiver Operating Characteristic (ROC) analysis was used to determine the prediction ability of ALP for osteoporosis, the maximum Youden’s index was used to determine the ALP cut-off value for osteoporosis. All data processing and analysis were conducted using statistical software SPSS 24.0 or STATA 11.0. Statistical significance level is α=0.05.

## Results

### Characteristics of subjects

A total of 12, 835 participants (female: 45.5%) were included, 1, 216 of whom (9.5%) were found to have osteoporosis based on dual X-ray detection and 1, 2712(99.04%) of whom had clinic normal ALP. People with osteoporosis had higher ALP levels than their counterparts without the condition (P < 0.001). Additionally, individuals with osteoporosis were more likely to be female, older, and of lighter body weight. However, they had a significantly higher waist-to-hip ratio than their typical peers (all p < 0.05). Furthermore, osteoporosis was associated with higher SBP, blood sugar, TC, and HDL levels compared to the standard controls. However, osteoporosis was not likely to be associated with fatty liver disease and had lower liver enzymes, such as AST and ALT. The UA was also lower in osteoporosis. Between the osteoporosis and control groups, DBP, TG, and LDL levels were not different (P > 0.05). The characteristics of the subjects are shown in **Table 1**.

**Table 1:** Characteristics of participants by osteoporosis status (Mean ± SD)*.

Variable	Non- osteoporosis N=11619	Osteoporosis N=1216	p
ALP (IU/L)	71.61 ± 22.8	80.44 ± 23	<0.001
Sex (female)	5099 (43.9)	737 (60.6)	<0.001
Age (year)	48.66 ± 10.58	60.55 ± 11.63	<0.001
Height (cm)	163.96 ± 8.31	159.23 ± 9.17	<0.001
Weight (kg)	65.44 ± 11.62	60.19 ± 11.32	<0.001
BMI	24.23 ± 3.16	23.63 ± 3.28	<0.001
Waist (cm)	82.32 ± 9.83	81.56 ± 9.8	0.015
Hip (cm)	94.81 ± 6.01	93.25 ± 6.4	<0.001
Waist-hip ratio	0.867 ± 0.07	0.873 ± 0.07	0.006
SBP (mmHg)	123.04 ± 17.45	129.66 ± 19.1	<0.001
DBP (mmHg)	74.83 ± 11.64	74.43 ± 11.47	0.263
GLU (mmol/L)	5.33 ± 1.4	5.47 ± 1.47	0.005
TC (mmol/L)	5.18 ± 0.98	5.33 ± 1.02	<0.001
TG (mmol/L)	1.81 ± 1.81	1.70 ± 1.38	0.072
HDL (mmol/L)	1.38 ± 0.32	1.45 ± 0.35	<0.001
LDL (mmol/L)	2.85 ± 0.76	2.85 ± 0.76	0.830
UA (µmoL/L)	349.54 ± 93.57	336.38 ± 93.89	<0.001
AST (U/L)	25.99 ± 22.81	22.98 ± 12.13	<0.001
ALT (U/L)	24.33 ± 19.23	22.87 ± 11.22	0.009
Fatty liver (Yes)	3328 (28.6)	264 (21.7)	<0.001

*Data were presented as mean+/-SD or N (%). ALP, alkaline phosphatase; BMI, body mass index; SBP, systolic blood pressure; DBP, diastolic blood pressure; GLU, blood glucose; GHB, glycated hemoglobin; TC, total cholesterol; TG, triglycerides; HDL, high-density lipoprotein cholesterol; LDL, low-density lipoprotein cholesterol; UA, uric acid; AST, aspartate aminotransferase; ALT, alanineaminotransferase.

### The relationship between ALP and osteoporosis

Five models were constructed to examine the relationship between ALP and osteoporosis. In Model 1, the odds ratio (OR) and 95% confidence interval (CI) were 0.016 (1.012-1.020; P <0.001), which indicated that the risk of osteoporosis was increased for every unit rise in ALP. The positive relationship persisted in the model, remaining significant even after adjusting for all potential confounders in the logistic regression analysis (all P < 0.001). The results of the regression analysis are shown in **Table 2**.

**Table 2:** The relationship between ALP and osteoporosis by logistic regression.

Model	B	se	P	OR of osteoporosis	95%CI of OR
Model 1	0.016	0.002	<0.001	1.016	1.012-1.020
Molde 2	0.012	0.002	<0.001	1.012	1.008-1.015
Model 3	0.012	0.002	<0.001	1.012	1.008-1.016
Model 4	0.012	0.002	<0.001	1.012	1.008-1.016
Model 5	0.013	0.002	<0.001	1.013	1.009-1.017

OR, odds ratio, Model1, unadjusted; Model 2, adjusted by age and sex; Model 3, model2 plus BMI and waist-hip ratio; Model4, model 3 plus blood pressure, GLU, TC, TG, HDL, LDL and UA,. Model 5, model 4 plus AST, ALT and fatty liver.

### The non-linear relationship between ALP and osteoporosis

**Figure 1:**
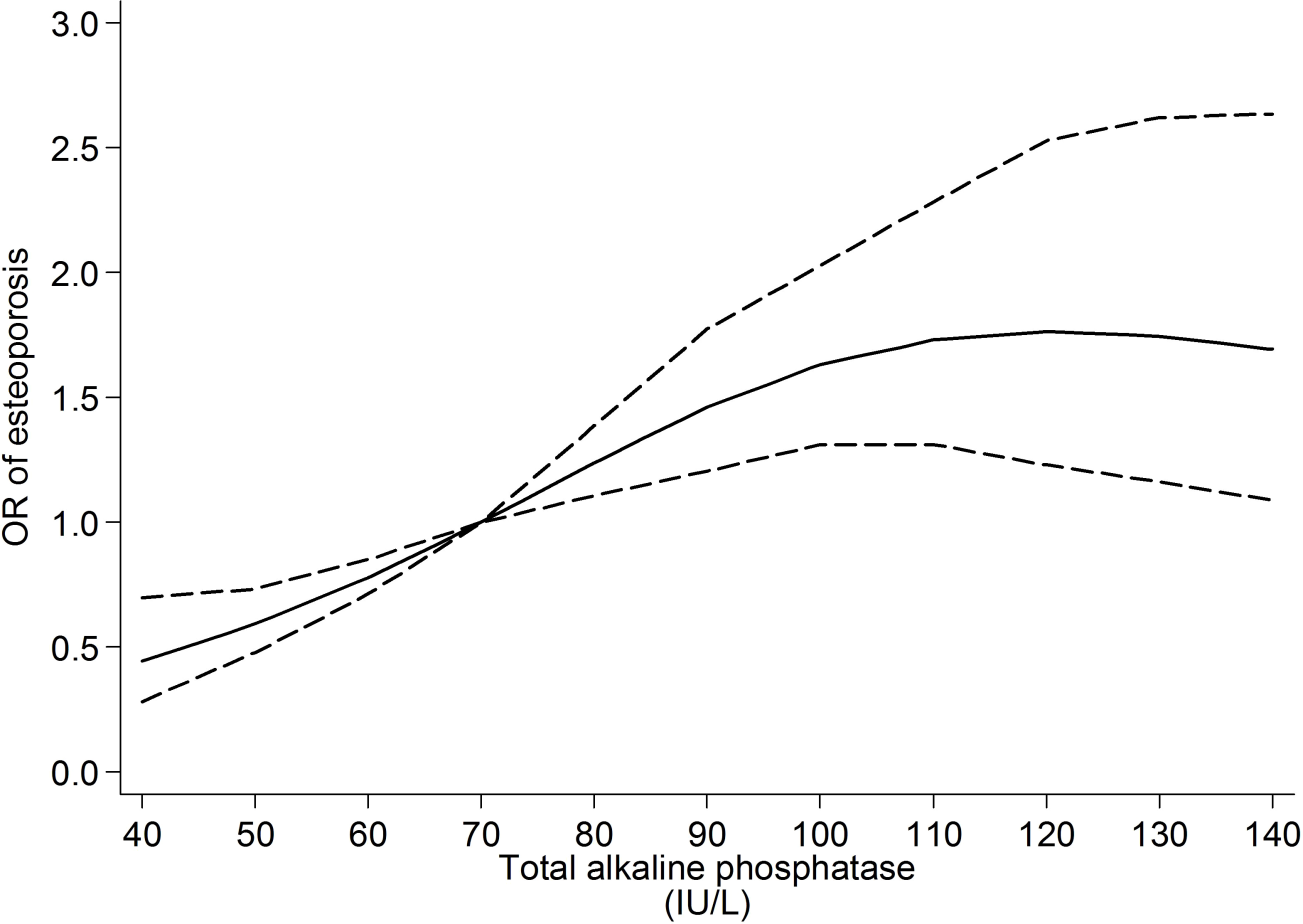
Relationship between ALP and the risk osteoporosis with restricted cubic spline regression. The median ALP (70IU/L) was set as reference value.

Restricted cubic spline regression suggested that the positive relationship between ALP and risk of osteoporosis was roughly linear. When it exceeded 100, the relationship between ALP and the risk of osteoporosis was attenuated, and the line became flatter, as shown in **Figure 1**.

### The ROC analysis of ALP and osteoporosis

**Figure 2:**
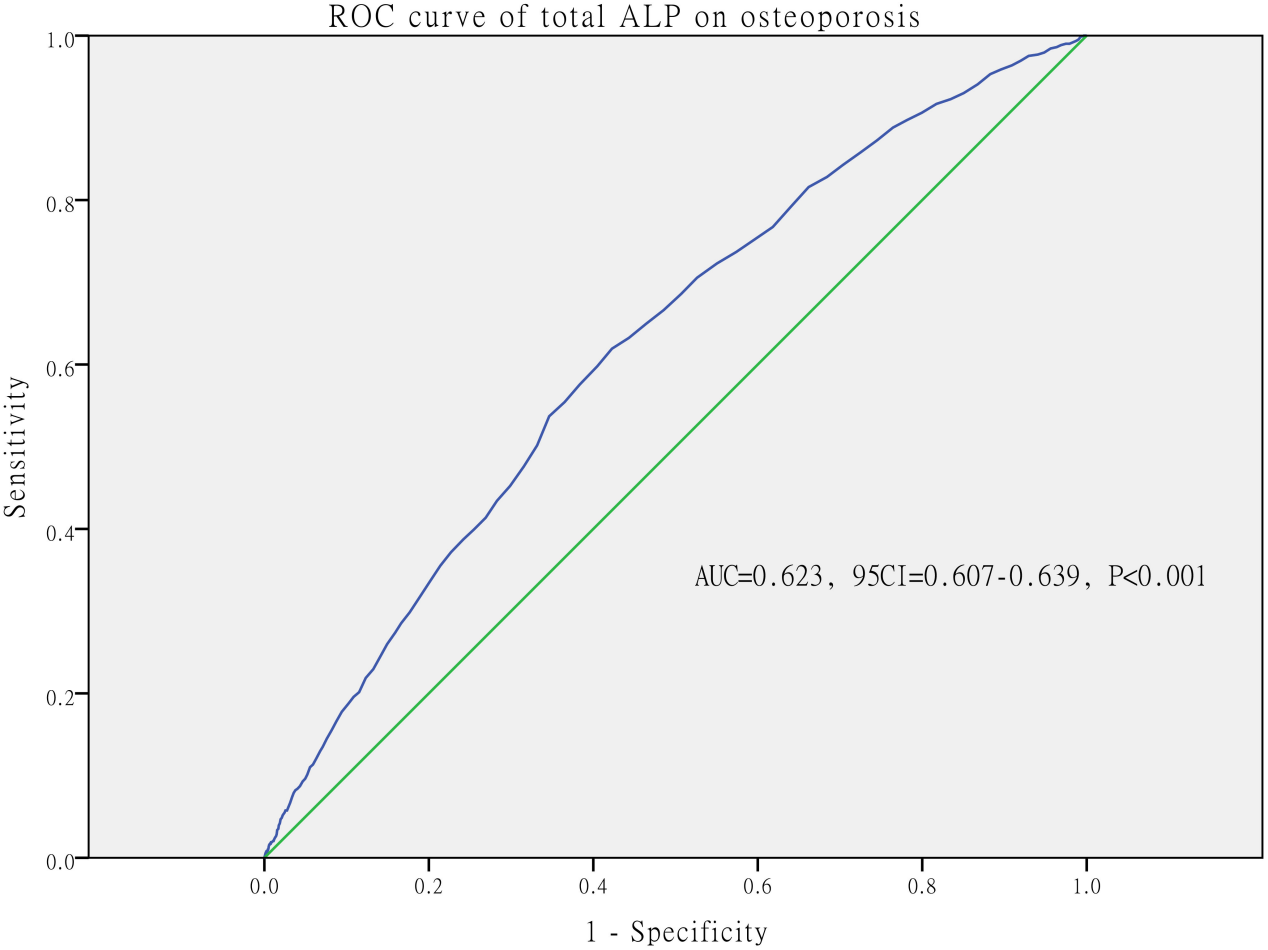
ROC analysis between total alkaline phosphatase (ALP) and osteoporosis in whole participants.

The ROC analysis was used to determine the discriminative ability of ALP for osteoporosis. The area under the ROC curve was 0.623 (P<0.001) and significantly greater than the reference line, as shown in **Figure 2**. The cut-off value for ALP to predict osteoporosis was 72UI/L based on the maximum Youden’s index of ROC curve.

### Subgroup analysis

We conducted a sensitive analysis across various subgroups. The relationship between ALP and the risk of osteoporosis was stronger when ALP was < 100 in females, who were younger, heavier, and healthier than their counterparts. When liver enzymes were abnormal (greater than 40 U/L), the relationship between ALP and osteoporosis was no longer evident. In subgroups with abnormal blood sugar and lipids, such as TC, TG, HDL, and LDL, the relationships between ALP and osteoporosis also become weak or disappear, as shown in **Table 3**.

**Table 3:** the subgroup analysis of the relationship between ALP and osteoporosis.

Variable	B	se	P	OR of osteoporosis	95%CI of OR
Alp					
<=100(U/L)	0.025	0.002	<0.001	1.025	1.021-1.030
>100(U/L)	0.001	0.002	0.739	1.001	0.997-1.005
Sex					
Female	0.020	0.002	<0.001	1.020	1.017-1.024
Male	0.007	0.002	0.002	1.007	1.003-1.011
Age					
<=50 year	0.015	0.003	<0.001	1.015	1.009-1.020
50–70 year	0.009	0.002	<0.001	1.009	1.006-1.012
>70 year	0.012	0.004	0.005	1.012	1.004-1.020
BMI					
<=24	0.015	0.002	<0.001	1.015	1.012-1.019
<=28	0.015	0.001	<0.001	1.015	1.012-1.018
>28	0.020	0.004	<0.001	1.02	1.012-1.029
Waist-Hip Ratio					
<=0.9	0.017	0.002	<0.001	1.017	1.014-1.02
>0.9	0.012	0.002	<0.001	1.012	1.007-1.017
Sbp					
<=130(mmHg)	0.014	0.002	<0.001	1.014	1.011-1.018
>130(mmHg)	0.012	0.002	<0.001	1.012	1.008-1.016
GLU					
<=5.6(mmol/L)	0.016	0.001	<0.001	1.016	1.013-1.019
>5.6(mmol/L)	0.009	0.004	0.010	1.009	1.002-1.016
TC					
<=6.2(mmol/L)	0.018	0.002	<0.001	1.018	1.015-1.022
>6.2(mmol/L)	0.011	0.002	<0.001	1.011	1.008-1.015
TG					
<=1.7(mmol/L)	0.022	0.002	<0.001	1.023	1.019-1.026
>1.7(mmol/L)	0.004	0.002	0.033	1.004	1.000-1.007
HDL					
<=1(mmol/L)	0.004	0.003	0.086	1.004	0.999-1.009
>1(mmol/L)	0.015	0.001	<0.001	1.016	1.013-1.018
LDL					
<=3.4(mmol/L)	0.016	0.002	<0.001	1.016	1.013-1.019
>3.4(mmol/L)	0.009	0.003	0.001	1.009	1.004-1.015
UA					
<=420(µmoL/L)	0.016	0.002	<0.001	1.016	1.013-1.019
>420(µmoL/L)	0.017	0.003	<0.001	1.017	1.010-1.024
AST					
<=40(U/L)	0.019	0.001	<0.001	1.019	1.016-1.022
>40(U/L)	0.003	0.002	0.077	1.003	1.000-1.007
AST					
<=40(U/L)	0.018	0.001	<0.001	1.019	1.016-1.021
>40(U/L)	0.003	0.002	0.101	1.003	0.999-1.007
Fatty liver					
No	0.016	0.001	<0.001	1.016	1.013-1.019
Yes	0.014	0.003	<0.001	1.014	1.008-1.019

ALP, alkaline phosphatase; BMI, body mass index; SBP, systolic blood pressure; DBP, diastolic blood pressure; GLU, blood glucose; GHB, glycated hemoglobin; TC, total cholesterol; TG, triglycerides; HDL, high-density lipoprotein cholesterol; LDL, low-density lipoprotein cholesterol; UA, uric acid; AST, aspartate aminotransferase; ALT, alanineaminotransferase.

## Discussions

In this study, we investigated the relationship between serum total ALP and osteoporosis in a health examination population of Chinese individuals with multiple confounders and multiple methods. The study revealed that the total ALP level in serum was significantly higher in patients with osteoporosis compared to those without osteoporosis. The relationship between ALP and osteoporosis persisted in multivariate analysis. In subgroup analysis, the relationships between ALP and osteoporosis were stronger in younger females and populations with good metabolic health. ALP had a weak but significant predictive ability for osteoporosis in the ROC analysis, with an AUC lower than 0.7, indicating poor discrimination (16).

Because the osteoporosis was diagnosed based on bone mass density measured by dual energy X-ray absorptiometry (DXA) at any sites of spine or hip in this study, the results implied that elevated ALP was directly related to decreased bone mass; the similar results were found in in a study that included US young adults aged 20–40 years. The same relationship between ALP and bone mass was observed in a health examination population of Chinese. Regarding osteoporosis, we found that the 1-unit increased ALP levels lead to a 1% increase in the prevalence of osteoporosis. Compared to a study that included US adults aged 18–85 from communities, in which a 1-unit increase in total ALP was associated with a 0.5% higher prevalence of osteoporosis (17), which was lower than that in our study. This difference may be due to the different sources of the participants.

The total ALP in serum was composed of bone ALP and liver ALP at a ratio of approximately 1:1 (18). The relationship between ALP and bone mass density or osteoporosis was due to the high correlation between total ALP and bone ALP. Therefore, the total ALP can represent the effects of bone ALP to some degree. However, this correlation may vary in certain conditions, especially when cells are damaged or injured. For example, in the high AST (> 40 U/L) and high ALT (> 40 U/L) groups, we found that the relationship between ALP and osteoporosis became insignificant. When liver cell injury occurs, the ALP doubles its usual level (19), resulting in more liver ALP being released into the blood. This may dilute the bone ALP and change the correlation between bone ALP and total ALP, making the relationship between total ALP and bone undetectable. We found that when total ALP exceeded 100, the relationship between ALP and osteoporosis also disappeared in the subgroup analysis. This may indicate that when ALP is much higher than a specific level, such as 100 in this study, the incremental portion of ALP may be due to liver cell injuries. In fatty liver subgroups, the changes were similar but not obvious, indicating that fatty liver itself does not imply serious injury to liver cells. In a study that compared the bone ALP and total ALP, it was found that in the presence of liver disease, the bone ALP was more sensitive to discriminate metabolic bone diseases (20).

Because total ALP, which stands for bone ALP, is a marker of bone formation during bone turnover, it means that accelerated bone turnover, rather than slowed bone turnover, is involved in the loss of bone mass. Regarding this phenomenon, available evidence suggests that increased serum total ALP in individuals with osteoporosis may be a compensatory response to declining bone mass density, which serves as a signal potentially promoting osteogenesis and reversing osteoporosis. The elevated bone ALP/total ALP in osteoporosis may confirm the change of bone ALP (21).

In subgroup analysis, we also found that in participants with abnormal metabolic indexes, such as blood sugar and lipids, the relationship between ALP and osteoporosis tended to disappear. In some studies, metabolic indices were found to be low and negatively related to bone loss and the occurrence of osteoporosis, findings similar to those in our study. Because metabolic symptoms were found to be related to impaired liver function (22), which may increase the liver ALP portion of total ALP, on the other hand, elevated ALP has also been reported to relate positively to metabolic disease (23). Under this condition, the only plausible explanation is that metabolic disease such as abnormal blood sugar and lipids may simultaneously raise the liver ALP and bone ALP, but with more liver ALP than bone ALP which change the normal ratio of liver ALP to the bone ALP and attenuate the relationship between total ALP and bone mass or osteoporosis. However, this presumption about the relationship between ALP and osteoporosis in metabolic diseases needs further studies to clarify in the future.

In clinic, ALP is measured for adjuvant diagnosis of some severe health conditions which may have significantly increasing of ALP. But in this study, all participants were apparently healthy and most of them(99%) had ALP in normal range. Therefore, the usual clinic reference value of ALP does not consider the abnormality of bone metabolism and is not suitable for healthy people in the risk evaluation of osteoporosis. For an apparently healthy person, the preliminary result of 72 IU/L of ALP in this study may be considered as a tentative threshold for initiating further bone health counseling in regular health management, including bone mass evaluation, lifestyle investigation and fracture risk assessment by the FRAX^®^ model.

This study has several strengths. First, the sample size is big, which makes the result stable and reliable. Second, in this study, we investigated the relationships between ALP and osteoporosis by considering multiple factors beyond those examined in other studies. Third, the data come from medical records, which are more accurate than self-reported information and are free from recall bias.

At the same time, several limitations should be noted. First, this is a cross-sectional study, the causal relationship between ALP and osteoporosis can not be confirmed. Second, this is a single-center study; the population in this center may introduce some bias, and the results may not be generalized to other populations. Third, some confounders such as malnutrition, medications, thyroid function, dietary pattern, low-energy fractures and physical activity, were not available in this study, which may have biased the results of our study.

## Conclusions

Based on our findings, the serum total ALP persistently and negatively relates to the risk of osteoporosis in a general population when ALP is clinic normal. The value of 72 IU/L serum total ALP can be considered as an tentative reference value for initiating further bone health counseling in health management. Further cohort studies are warranted to evaluate the role of ALP in osteoporosis or fracture risk prediction.

## Data Availability

The raw data supporting the conclusions of this article will be made available by the authors, without undue reservation.
